# Synergistic protective effects of curcumin nano-emulsion and virgin coconut oil against thermal oxidation of sunflower oil and its hepatorenal toxicity in rats

**DOI:** 10.3389/fnut.2026.1864714

**Published:** 2026-07-07

**Authors:** Seham E. Almasoudi, Nawal A. Ozaybi, Eman S. Alamri, Hala M. Bayomy, Fayza M. El Ezaly

**Affiliations:** 1 Department of Food Science and Nutrition, Faculty of Science, University of Tabuk, Tabuk, Saudi Arabia; 2 Department of Home Economics, Faculty of Specific Education, Mansoura University, Mansoura, Egypt

**Keywords:** heated oil toxicity, hepatotoxicity, nano-curcumin, oxidative stress, synergistic effect, virgin coconut oil

## Abstract

**Introduction:**

Repeated heating of vegetable oils leads to the generation of toxic oxidation products, which activate systemic oxidative stress and cause multi-organ injuries. This study examined the hepatorenal synergistic protection of Curcumin Nano-Emulsion (CNE) and virgin coconut oil (VCO) against the toxicity of thermally stressed sunflower oil (HSO) and explored the underlying antioxidant and anti-inflammatory mechanisms.

**Methods:**

CNE was characterized by nanoscale particle size, high encapsulation efficiency, and excellent stability. HSO was supplemented with VCO (20% w/w) and CNE (200 ppm) to evaluate improvements in oxidative stability *in vitro* by measuring peroxide value, p-anisidine value, total polar compounds, and toxic aldehydes. For the in vivo study, sixty-four male Wistar rats were fed different oil-based diets for two months. Biochemical parameters, oxidative stress markers, inflammatory indices, and histopathological changes in liver and kidney tissues were assessed. Immunohistochemical analysis was performed to evaluate NF-κB and Nrf2 signaling pathways.

**Results:**

Supplementation of HSO with VCO and CNE significantly improved oxidative stability compared with individual treatments and TBHQ, with marked reductions in peroxide value, p-anisidine value, total polar compounds, and toxic aldehydes. HSO induced significant hepatorenal injury, dyslipidemia, oxidative stress, and inflammation in rats. The combined treatment (HSO + VCO + CNE) markedly ameliorated these alterations, nearly restoring biochemical parameters to normal levels and minimizing histopathological damage. A strong downregulation of the NF-κB pathway and activation of the Nrf2 antioxidant pathway were observed.

**Discussion:**

Co-administration of curcumin nano-emulsion and virgin coconut oil effectively enhances the oxidative stability and safety of thermally stressed edible oils. These findings suggest a promising natural strategy to reduce oil-induced toxicity and support safer industrial oil processing approaches aligned with public health protection.

## 1 Introduction

Because the world depends heavily on both domestic and commercial vegetable oils for frying and food processing, understanding their chemical stability under heat stress is crucial. Constant reheating is the normal practice in most households and working places, therefore, the oils undergo a chain of degradation reactions such as auto-oxidation, polymerization, and hydrolysis (1). These processes generate both the primary and the secondary oxidation products such as hydroperoxides, aldehydes, ketones (e.g., malondialdehyde and hexanal) are accompanied by very high-molecular-weight degradation compounds like the Total Polar Compounds (TPC) (2). Consumption of these thermally damaged oils is a major cause of cardiovascular diseases, cancer, metabolic disorders, and may result in severe organ damages – especially liver and kidneys, primary due to systemic oxidative stress and chronic inflammation (3, 4).

One of the major bottlenecks in limiting the adverse effect of heated oils is in the antioxidant arsenal where the antioxidants not only effectively neutralize the radicals but are also entirely safe and highly heat-stable to still function during cooking and storage. Nevertheless, some synthetic antioxidants like tert-butylhydroquinone (TBHQ) have been met with an increasing wave of regulatory and safety concerns despite being most used for food applications (5). As an outcome, the attention has been firmly focused on natural alternatives possessing high antioxidant activity.

Curcumin, the main bioactive curcuminoid, has been isolated from *Curcuma longa* and is known for its powerful antioxidant, anti-inflammatory, and hepatoprotective properties (6). Nevertheless, its use remains compromised mainly because of its low water solubility, poor physicochemical stability, and fast metabolic clearance causing extremely low bioavailability (7). Nano-encapsulation is considered as a remarkably effective medium through which curcumin carriers could overcome their drawbacks as CNE formulations lead to a marked increase in solubility, stability as well as biological availability of curcumin (8).

Virgin coconut oil (VCO) is one more natural product to feature a health-promoting side of its nature that is extensively studied. VCO is a significant source of medium-chain triglycerides, primarily lauric acid, and it has bioactive minor components like tocopherols and polyphenols. VCO not only has an everlasting intrinsic oxidative activity and shows anti-inflammatory properties but also it is more resistant to the oxidation compared to the oils rich in polyunsaturated fatty acids (9). There is also a study that reveals that VCO can alleviate chemically induced hepatic and renal toxicity by modulating the oxidative and inflammatory pathways. It is well established that the potential individual protective effects of nano-curcumin and VCO have already been extensively studied. However, their combined effect as a whole-treated thermally stressed vegetable oil system has not yet been explored. As far as we can remember, there is no available scientific research that subsequently proves the synergism of CNE and VCO in the oxidative stability enhancement of the sunflower oil that has been thermally treated and its systemic toxicity reduction that is in vivo (10). This is of foremost importance since the leaders in the field of oxidative damage and systemic toxicity are completely complementary mechanisms—CNE radical-scavenging capacity and VCO bioactive compounds and inherently oxidative stability—which can theoretically render a more efficient defense against lipid peroxidation and systemic harm.

Sunflower oil was selected as the model system for studying thermal-induced degradation. Therefore, the objectives of this study were: (1) to prepare and characterize a stable curcumin nano-emulsion; (2) to assess its synergistic antioxidant effect with VCO at thermal stress conditions; and (3) to reveal the hepatorenal protective mechanisms in a rat model.

It is through these various research approaches however that the authors aim establish a solid scientific basis of a natural, safe, and effective intervention that is capable of enhancing the stability and nutritional quality of the daily cooking oils. This strategy is designed to promote healthier eating habits and form a foundation for potential future industrial applications.

## 2 Materials and methods

### 2.1 Materials

Curcumin powder (≥94% purity) was purchased from Sigma-Aldrich (Salah Salem St., Cairo, Egypt). Refined high-linoleic sunflower oil (SO) and Virgin Coconut Oil (VCO) were procured from a local market (Mansoura, Egypt). Tween 80 [Polyoxymethylene (20) sorbitan monooleate] and Span 80 (Sorbitan monooleate) were obtained from Sigma-Aldrich (Salah Salem St., Cairo, Egypt), TBHQ was a food system as it is rich in polyunsaturated fatty acids, is a common staple in households, and is very susceptible to oxide-grade product with a purity of 99% or more, obtained from Chemitex Egypt for Trading & Agencies S.A.E (Alexandria, Egypt). All other chemicals and reagents used in this study were of analytical or high-performance liquid chromatography (HPLC) grade.

### 2.2 Preparation and characterization of curcumin nano-emulsion (CNE)

#### 2.2.1 Preparation for nano-emulsion

A stock solution of curcumin was prepared by dissolving it in the carrier oil (refined sunflower oil was selected as the carrier oil) at a concentration of 10 mg/mL. The oil phase, containing the curcumin stock solution and Span 80 (co-surfactant), was slowly mixed with the aqueous phase, containing Tween 80 (surfactant) and deionized water, under continuous magnetic stirring at 500 rpm for 30 min to form a coarse emulsion. The final ratio of oil phase: surfactant: co-surfactant: water was optimized to achieve maximum stability and encapsulation efficiency.

To avoid overheating, the crude emulsion was then high-intensity sonicated (High-intensity probe sonication, Branson Sonifier SFX550, Emerson, United States) with a 1/2-inch probe tip at 40% amplitude in 10 min (with 5-s pulses and 5-s cooling periods in an ice bath). This was what resulted in the product Curcumin Nano-Emulsion (CNE). The CNE was packed in amber glass bottles at 4 °C to be used (11).

#### 2.2.2 Characterization and stability of nano-emulsion

*Particle size, polydispersity index (PDI), and zeta potential:* Dynamic Light Scattering (DLS) was used to measure the mean hydrodynamic diameter and PDI of the CNE with a Zetasizer Nano ZS (UK, Malvern). The deionized water was added (1:100) to the samples to obtain an optimal level of scattering. To determine the stability of the nano-emulsion electrostatic stability, the Zeta Potential (ζ) was determined. Measurement was done in three repetitions at 25 °C (12).

*Encapsulation efficiency (EE%):* The determination of the EE% has been done using the indirect approach. In brief, the unencapsulated curcumin was ultrafiltered by centrifugation (5000 × g) at 30 minutes on ultrafiltrate at 420 nm, based on a previously prepared standard sample (13). The concentration of the free curcumin in the filtrate was measured with the usage of the UV-vis spectrophotometer (Shimadzu, Japan) at 420 nm. The equation used to calculate the EE% was as follows:


Encapsulation Efficiency(EEpercentage)=(Total Curcumin−

Free Curcumin)/Total Curcumin)×100.

*Morphology (transmission electron microscopy—TEM)*: The microstructure and morphology of Curcumin Nano-Emulsion (CNE) was studied under Transmission Electron Microscopy (TEM) (JEOL JEM-1010, Japan). One drop of the diluted CNE was put on the carbon-coated copper grid and stained with a 2% solution of phosphotungstic acid within a 60 s time interval and left to dry at room temperature and finally imaged (14).

*Nano-emulsion stability:* The stability of the CNE was assessed by storing it at 4°C for 8 weeks (the duration of the in vivo study). Particle size, PDI, and zeta potential were measured weekly. The CNE was considered stable if no significant changes (*p*> 0.05) in these parameters and no visible signs of phase separation or precipitation occurred during the storage period (15).

### 2.3 Thermal oxidation protocol and *in vitro* experimental groups

#### 2.3.1 Heating protocol

Refined high-linoleic sunflower oil (SO) was exposed to an intensive thermal stress protocol to mimic commercial deep-frying situations. The oil was heated in open stainless-steel containers (1 L capacity) through a thermostatically controlled electric fryer at 180 ± 5 °C for 8 h per day over 3 consecutive days. The oil was taken back to room temperature overnight. Oil samples were taken every 4 h during the heating period for chemical analysis. The oil obtained was named Heated Sunflower Oil (HSO).

#### 2.3.2 Justification of dosages

The Virgin Coconut Oil (VCO) (20% w/w) and Curcumin Nano-Emulsion (CNE) (200 ppm curcumin equivalent) concentration was determined through an extensive review of the available literature and pilot studies performed in our laboratory. It has been shown that VCO concentrations from 10 to 20% give substantial protection against lipid oxidation with no change in the organoleptic characteristics of the oil (16). The 200-ppm concentration for curcumin was decided upon because it is a standard and effective dose in similar *in vivo* studies, offering a compromise between therapeutic efficacy and safety, and at the same time avoiding possible pro-oxidant effects at higher concentrations (17).

#### 2.3.3 *In vitro* experimental groups

*In vitro* antioxidant activity was evaluated in the six treatments of heated sunflower oil (Table 1).

**TABLE 1:** The HSO was divided into six groups for *in vitro* antioxidant evaluation.

Group	Treatment	Description
SO	Unheated control	Unheated Sunflower Oil (Control)
HSO	Negative control	Heated Sunflower Oil (HSO)
HSO+VCO	Single treatment	HSO supplemented with Virgin Coconut Oil (VCO) at 20% (w/w)
HSO+CNE	Single treatment	HSO supplemented with Curcumin Nano-Emulsion (CNE) at a concentration equivalent to 200 ppm of Curcumin
HSO+VCO+CNE	Synergistic treatment	HSO supplemented with VCO (20% w/w) and CNE (200 ppm Curcumin)
HSO+TBHQ	Positive control	HSO supplemented with the synthetic antioxidant TBHQ at 200 ppm

### 2.4 Chemical analysis of oil samples

The following parameters were measured on the oil samples collected from the *in vitro* experiment:

#### 2.4.1 Primary oxidation products

The peroxide value (PV) of the oil samples was determined following the (18) Official Method Cd 8–53. Briefly, an accurately weighed oil sample (≈ 5.0 g) was dissolved in a chloroform–acetic acid mixture (3:2, v/v), followed by the addition of saturated potassium iodide (KI). The liberated iodine was allowed to react in the dark for 1 min, then titrated with standardized sodium thiosulfate solution (0.01 N) using starch indicator. The PV was calculated and expressed as milliequivalents of active oxygen per kilogram of oil (meq O2/kg).

#### 2.4.2 Secondary oxidation products

##### 2.4.2.1 p-Anisidine value (p-AV)

The p-anisidine value was determined according to AOCS Official Method Cd 18–90 (19). Briefly, the oil sample was dissolved in iso-octane, and the absorbance was recorded at 350 nm before and after reaction with p-anisidine reagent.

##### 2.4.2.2 Advanced oxidation products

A Testo 270 cooking oil tester (Testo Inc., Germany) was used to determine the total polar compounds (TPC). Before making any measurement, the device was calibrated with the help of the reference oil supplied by the manufacturer to guarantee that the analysis will be accurate and reproducible. To minimize the threat of thermal variability, oil samples were mixed gently and then measured within a controlled temperature range as specified by the manufacturer (about 40–50 °CC). Measurements were done according to the standardized operating protocol, and TPC values were taken in percentage (%) of total polar materials. Quality control measures were conducted regularly during the analysis to ensure stability and consistency of the instruments (20).

##### 2.4.2.3 Advanced analysis of volatile compounds

Gas chromatography-mass spectrometry (GC-MS): This is utilized in the identification and determination of selected toxic volatile aldehydes, such as Hexanal, trans-2-Nonanal, and Acrolein. The technique was a dynamic headspace solid-phase microextraction (HS-SPME) with the subsequent GC-MS analysis on an Agilent 7890B GC system with a 5977A MSD (Agilent Technologies, United States) device (21).

### 2.5 Experimental design and animal study

#### 2.5.1 Animals and ethical approval

A total of 64 male albino rats (Wistar strain), weighing 180–220 g, were obtained from the Animal House of the Faculty of Pharmacy, Mansoura University, Egypt. The animals were housed in stainless-steel cages under controlled laboratory conditions (temperature: 22 ± 2 °C, relative humidity: 60 ± 5%, and a 12-h light/dark cycle). They were acclimatized for 1 week before the start of the experiment, followed by an 8-week experimental period. Throughout the study, the rats had free access to a standard basal diet formulated according to the National Research Council (NRC, 1995) guidelines and water *ad libitum*. All animal procedures were performed strictly in accordance with the guidelines of the Mansoura University–Animal Care and Use Committee MU-ACUC (OTH.R.25.12.8)

#### 2.5.2 Animal grouping and treatment

The animals were randomly allocated into eight experimental groups according to the type of oil and antioxidant treatment administered as detailed in Table 2. The Normal Control group (G1) received a standard diet with unheated sunflower oil (SO), whereas the Toxic Control (G2) was fed heated sunflower oil (HSO) without additives. Groups G3–G6 received HSO supplemented with different antioxidant interventions: virgin coconut oil at 20% (w/w) (G3), curcumin nano-emulsion equivalent to 200 ppm curcumin (G4), and TBHQ at 200 ppm (G6), while G5 received the combined VCO (20% w/w) and CNE (200 ppm) treatment to assess potential synergistic effects. Two additional control groups were included: VCO Control (G7), receiving unheated SO + 20% VCO, and CNE Control (G8), receiving unheated SO + 200 ppm curcumin, these groups were specifically designed using unheated SO. This setup was intentionally integrated to evaluate the independent physiological effects and to rule out any inherent or potential Toxicity of Virgin Coconut Oil and Curcumin Nano emulsion on healthy liver and kidney tissues, thereby establishing a confirmed safety profile for both interventions

**TABLE 2:** Experimental grouping and dietary treatments administered to rats during the study period.

Group	Treatment	Description
G1	Normal Control (NC)	Standard diet + Unheated Sunflower Oil (SO)
G2	Toxic Control (TC)	Standard diet + Heated Sunflower Oil (HSO)
G3	HSO + VCO	Standard diet + HSO + VCO (20% w/w)
G4	HSO + CNE	Standard diet + HSO + CNE (200 ppm Curcumin)
G5	HSO + VCO + CNE	Standard diet + HSO + VCO + CNE (Synergistic Group)
G6	HSO + TBHQ	Standard diet + HSO + TBHQ (200 ppm)
G7	VCO Control	Standard diet + Unheated SO + VCO (20% w/w)
G8	CNE Control	Standard diet + Unheated SO + CNE (200 ppm Curcumin)

The selected concentrations of VCO, CNE, and TBHQ were based on published evidence indicating that VCO at 10–20% enhances oxidative stability and exerts hepatoprotective activity in thermally stressed oils (22) The 200 ppm dose of CNE corresponds to effective antioxidant and anti-inflammatory levels commonly used in *in vivo* toxicological studies (23). TBHQ was applied at 200 ppm, the maximum allowable level in edible oils according to international regulatory standards and a widely adopted reference dose in comparative antioxidant research (24).

The rats were divided randomly into eight groups (*n* = 8). The oil treatments were administration orally by gavage at a dose of 5 mL/kg body weight per day for 8 weeks.

### 2.6 Measurements and analyses

#### 2.6.1 Biometric measurements

After the experiment, rats were deprived of food for the whole night and lit with a ketamine/xylazine combination (ketamine 80 mg/kg and xylazine 10 mg/kg, intraperitoneally). This was done following the ethical rules for animal experimentation. At the time of sacrifice, blood samples were collected through cardiac puncture. Later, the liver and kidneys were gently removed, washed with saline, and fixed in 10% neutral buffered formalin at once for histopathological examination. The tissues fixed with formalin were cut for routine hematoxylin and eosin (H&E) staining and for an immunohistochemical (IHC) study.

##### 2.6.1.1 Liver functions

Serum levels of ALT concentration were measured using the Diamond Diagnostics, Egypt Kit (Cat. No EC2.6.1.1) with a semi-auto chemistry analyzer, based on an enzymatic colorimetric assay as described by Reitman and Frankel (25).

Serum levels of AST concentration were measured using the Diamond Diagnostics, Egypt Kit (Cat. No EC2.6.1.2) with a semi-auto chemistry analyzer, based on an enzymatic colorimetric assay as described by Tietz (26).

##### 2.6.1.2 Kidney functions

Serum urea concentration was measured using the Spectrum-Urea Kit (Egypt-IFUFCC40) with a semi-auto chemistry analyzer, based on a colorimetric assay for quantifying urea levels as described by Jing et al. (27).

Creatinine concentration in serum was determined using the Spectrum-Creatinine Kit (Egypt-IFUFCC72) with a Sesil spectrophotometer (England) and a semi-auto chemistry analyzer, based on the method described by Tietz (26).

Uric acid concentration in serum was measured using the Spectrum Diagnostics Uric Acid Kit (Egypt-IFUFCC46) with a Sesil spectrophotometer (England) and a semi-auto chemistry analyzer, based on the method described by Fossati and Prencipe (28).

##### 2.6.1.3 Lipid profile

Total cholesterol (TC), triglycerides (TG), high-density lipoprotein cholesterol (HDL-C), and low-density lipoprotein cholesterol (LDL-C) were measured using enzymatic colorimetric assays with commercial diagnostic kits (Bio diagnostic, Giza, Egypt), following the manufacturer’s instructions. The methods used for total cholesterol and triglycerides were those previously described by Allain et al. (29). HDL-C and LDL-C concentrations were subsequently calculated using the Friedewald formula as follows:


LDL−C=TC−(HDL−C+TG/5)⁢and

VLDL−C=TG/5⁢(30).

##### 2.6.1.4 Oxidative stress markers

After blood collection, serum was separated by centrifugation at 3,500 rpm for 10 min at 4°C and kept at -80°C. Liver and kidney tissues were washed with ice-cold saline, homogenized (10% w/v) in phosphate buffer (0.1 M, pH 7.4), and centrifuged at 10,000 × g for 15 min at 4°C. The serum and tissue supernatants obtained were subjected to the assay of MDA, H2O2, GSH, SOD, CAT, and GPx by using commercial kits (Bio diagnostic, Giza, Egypt) following the manufacturer’s instructions (31).

##### 2.6.1.5 Inflammatory markers

The levels of interleukin-6 (IL-6), tumor necrosis factor-alpha (TNF-α), and interleukin-1 beta (IL-1β) in rat serum were determined using rat-specific ELISA kits (R&D Systems, United States) according to the manufacturers’ instructions. Prostaglandin E2 (PGE2) levels were determined by a competitive ELISA kit specific for prostaglandins (Cayman Chemical, United States). Duplicate assays were done for all samples, and the absorbance was measured at 450 nm with a microplate reader (32).

#### 2.6.2 Histopathology and immunohistochemistry

##### 2.6.2.1 Histopathological examination (H&E)

Liver and kidney tissues were fixed, processed, and embedded in paraffin wax. Sections of 5 μm thickness were cut, stained with Hematoxylin and Eosin (H&E) (33) and examined under a light microscope. A blind pathologist evaluated 5–10 randomly selected high-power fields (HPFs, ×400 magnification) per section, using a semi-quantitative scoring system to evaluate the extent of histopathological damage. Changes were scored on a scale of 0 to 4 (0 = none, 1 = mild/ > 25% area affected, 2 = moderate/26–50% area affected, 3 = severe/51–75% area affected, 4 = very severe/75% < area affected) for key parameters including steatosis, necrosis, and inflammatory cell infiltration. The average damage score for each group was calculated (34).

**Immunohistochemical (IHC) Staining:** The liver and kidney sections were stained by using IHC to determine the expression of 4-Hydroxynonenal (4-HNE), Nuclear Factor-kappa B (NF-κB p65), Tumor Necrosis Factor-alpha (TNF- 65), and Nuclear factor erythroid 2-related factor 2 (Nrf2). Slides were deparaffinized, rehydrated and then antigen retrieved. The primary antibodies (anti-4-HNE, anti-NF- κB p65, anti-TNF-α, anti-Nrf2; Abcam, United Kingdom) were introduced overnight at 4 °C and the following next steps were conducted: incubation with the secondary antibody, development of the DAB chromogen. To quantify the protein expression, 5–8 random high-power fields ($\times$400) per section were captured, and ImageJ software with the “Color Deconvolution” plugin was used to quantify the expression as the mean percentage of positive staining relative to the total area (% Positive Staining Area) (NIH, United States). To assess pathway activation, Nrf2 and NF-κB nuclear localization were measured separately (32).

### 2.7 Statistical analysis

All data were expressed as the mean ± standard deviation (SD). Differences between groups were analyzed using One-Way Analysis of Variance (ANOVA) followed by Tukey’s *post-hoc* test. The level of statistical significance was set at *p* < 0.05. Statistical analyses were performed using GraphPad Prism (Version 9, GraphPad Software Inc., San Diego, CA, United States) and using Python (Google Colab environment).

## 3 Results

### 3.1 Characterization and stability of curcumin nano-emulsion (CNE)

Comprehensive physicochemical characterization of the Curcumin Nano-emulsion (CNE) resulted in achieving a stable CNE (Table 3). The CNE showed a nanoscale droplet size with a hydrodynamic diameter of 112.5 ± 4.3 nm measured using DLS with a polydispersity index of (0.18 ± 0.02), which is narrow and homogenous. It was also observed that the formulation exhibited high negative zeta potential, which indicated the electrostatic stability of the system. The efficiency of loading the curcumin into the nano-emulsion droplets is also displayed by the high encapsulation efficiency of the droplets.

**TABLE 3:** Physicochemical characterization of the curcumin nano-emulsion (CNE).

Parameter	Value
Mean particle size (nm)	112.5 ± 4.3
Polydispersity index (PDI)	0.18 ± 0.02
Zeta potential (mV)	-35.2 ± 1.5
Encapsulation efficiency (%)	92.4 ± 2.1

Data are expressed as Mean ± SD (*n* = 3).

**FIGURE 1:**
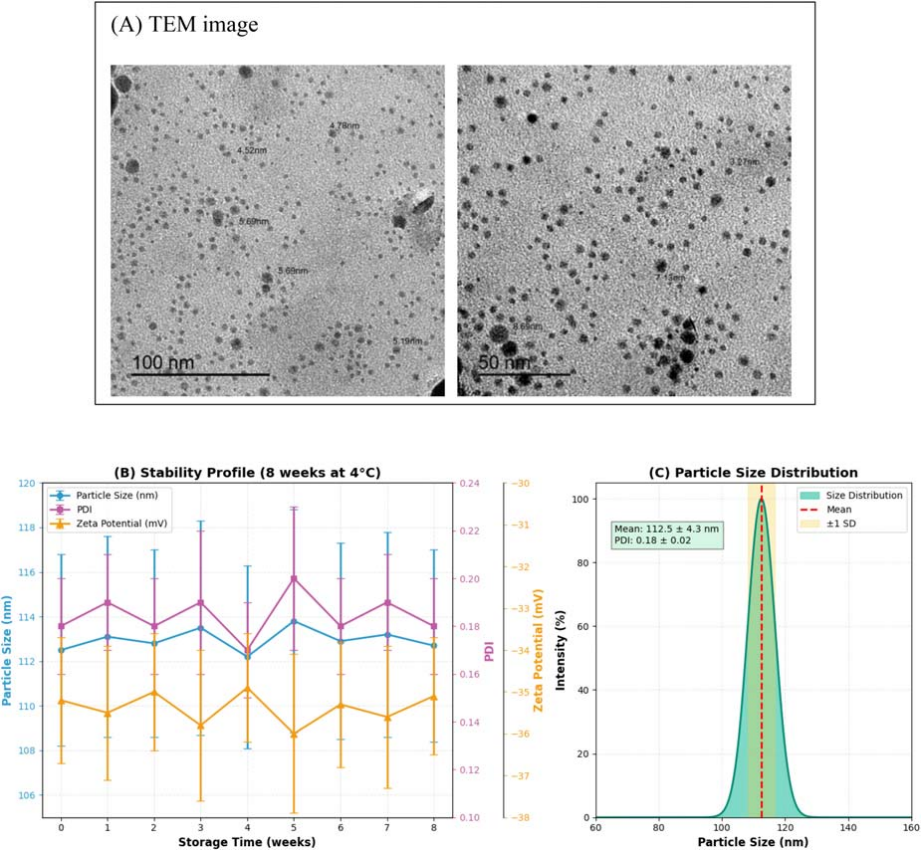
Characterization of curcumin nano-emulsion (CNE). **(A)** TEM image showing spherical and uniformly dispersed nanodroplets. **(B)** Stability profile of particle size, PDI, and zeta potential during 8 weeks of storage at 4 °C (no significant changes, *p* > 0.05). **(C)** Particle size distribution histogram showing a mean size of 112.5 ± 4.3 nm and PDI of 0.18 ± 0.02, indicating narrow size distribution and good homogeneity.

The presence of well dispersed spherical smooth nanodroplets without any evidence of aggregation was observed under TEM imaging (Figure 1A) and the size distribution curve of the particle (Figure 1C) showed that the vast majority of the particles were concentrated around the main peak (∼113 nm), which underlines the homogeneity of the formulation.

Stability analysis was done after 8 weeks at 4 °C (Figure 1B) and found no significant alteration in the particle size (*p* = 0.87), PDI (*p* = 0.92), or the zeta potential (*p* = 0.79). No creaming, phase separation, and visual instability were observed during the storage period, which proved the outstanding physical stability of CNE system.

### 3.2 *In vitro* antioxidant efficacy in thermally stressed oil

**FIGURE 2:**
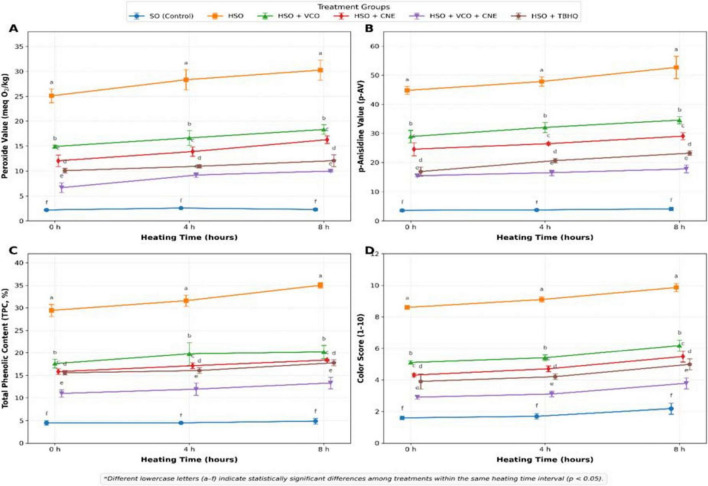
Comparative effects of different treatments on oxidative stability, phenolic content, color characteristics, and p-anisidine levels of sunflower oil during thermal stress. **(A)** Peroxide value (meq O_2_/kg). **(B)** p-Anisidine value (P-AV). **(C)** Total phenolic content (TPC, %). **(D)** Color score (L*, a*, b*).

Figure 2 gives a detailed analysis of how different treatments affect the physicochemical stability of sunflower oil exposed to repeated thermal exposure. All four panels depict variations in the main oxidative and quality indices of three heating periods (0, 4, and 8 h) of six experimental conditions, i.e., untreated sunflower oil (SO; Control), repeatedly heated sunflower oil (HSO), HSO with the addition of virgin coconut oil (HSO + VCO), nano-curcumin emulsion (HSO + CNE), its combination (HSO + VCO + CNE), and the synthetic antioxidant tert-butylhydroquinone (HSO).

Figure 2A shows that all samples heated showed a progressive change in the values of peroxide (PV) upwards, as would be expected of the primary oxidation products formed throughout the heating process. It is worth noting that the increase in PV was significantly reduced with oil treated with VCO or CNE or their combination with untreated HSO, indicating their protective actions of reducing hydroperoxides formation.

Figure 2B represents the overall phenolics content (TPC%), which decreased in the untreated heated oil, indicating the destruction of phenolics to heat stress. On the other hand, VCO-enriched and/or CNE formulations maintained higher levels of phenolics by a great margin, which is a sign of a higher thermal resilience due to the presence of antioxidant compounds as well.

Figure 2C displays the color score (1–10 scale), whereby thermal processing caused visible darkening (at a higher value) of HSO. VCO and CNE treatments, or a combination of the two, significantly reduced the breakdown of color indicating a decrease in pigment oxidation and polymerization.

Figure 2D shows the values of p-anisidine (p-AV), which is a measure of secondary oxidation products (aldehydes). HSO presented a strong increase in p-AV at long-term heating but treated oils and especially the VCO + CNE mixture showed significantly lower increments, which proved better inhibition of secondary oxidative degradation.

Altogether, the figure shows that natural antioxidant treatments, in particular, a synergistic mixture of VCO and nano-curcumin, have a great impact on the oxidative stability and thermal quality of sunflower oil in comparison to untreated heated oil and can perform similar or even better than TBHQ.

### 3.3 Mitigating heat-induced aldehyde formation in sunflower oil: GC–MS evidence of the protective roles of VCO and CNE

**FIGURE 3:**
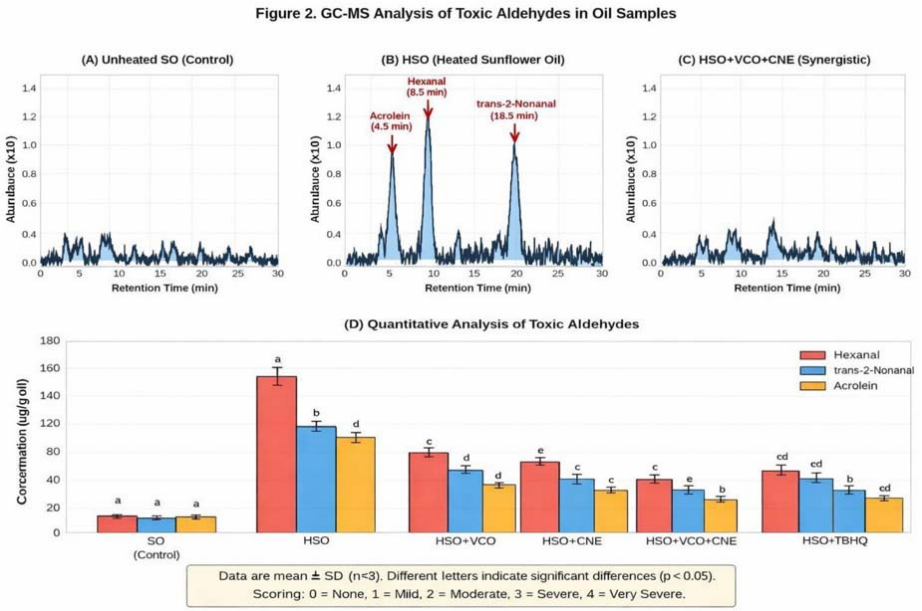
GC-MS analysis of volatile aldehydes in oil samples after 8 hours of thermal treatment. **(A)** Unheated sunflower oil (Control). **(B)** Heated sunflower oil (HSO), showing significant peaks of hexanal and trans-2-nonanal. **(C)** HSO treated with a synergistic combination (HSO+VCO+CNE), demonstrating a dramatic reduction in aldehyde peaks. **(D)** Quantitative bar chart of all treatment groups for hexanal, trans-2-nonanal, and acrolein (Data are mean ± SD, *n* = 3; different letters indicate significant differences at (*P* < 0.05).

Figure 3 analysis of GC–MS showed no formation of aldehydes in the unheated sunflower oil (SO), but a strong formation in acrolein, hexanal, and trans-2-nonenal in the heated sunflower oil (HSO) with distinct retention times, which were 4.5, 8.5, and 16.5 min, respectively. The introduction of virgin coconut oil (VCO) and Curcumin Nano-Emulsion (CNE) minimized the intensity of the aldehyde peaks, whereas the introduction of the two in combination (HSO + VCO + CNE) resulted in the greatest reduction, and it was almost equal to the values in control.

The quantitative analysis of the results proved that HSO had the highest levels of all the toxic aldehydes (*p* < 0.05). VCO and CNE treatment also reduced these levels significantly, with the combination of both treatments producing the best result. TBHQ minimized the occurrence of aldehydes too but not as much as the synergistic VCO+CNE. VCO and Curcumin Nano-Emulsion, especially in combination, exhibited good protective properties against heat-induced aldehyde production in sunflower oil.

### 3.4 *In vivo* assessment in rats

#### 3.4.1 Lipid profile parameters

Table 4 data on the lipid profile indicate evident changes in the experimental groups. HSO group (G2) had the strongest dyslipidemia with significantly higher TC, TG, LDL-C, and VLDL-C and lower HDL-C than all control groups. On the contrary, the VCO and Curcumin Nano-Emulsion control groups (G7 and G8), had normal lipid patterns like the standard control (G1).

**TABLE 4:** Effects of different oil treatments on serum lipid profile parameters in experimental groups.

Group	TC (mg/dL)	TG (mg/dL)	LDL-C (mg/dL)	HDL-C (mg/dL)	VLDL-C (mg/dL)
G1: SO (Control)	84.71^g^ ± 8.70	73.33 ^e^ ± 8.59	23.12 ^f^ ± 3.56	46.92 ^a^ ± 3.61	13.29 ^f^ ± 1.96
G2: HSO	197.29 ^a^ ± 16.54	155.75 ^a^ ± 12.31	141.63 ^a^ ± 11.20	24.51^h^ ± 2.26	32.19 ^a^ ± 4.40
G3: HSO+VCO	124.03 ^b^ ± 14.93	97.91 ^b^ ± 8.73	68.65 ^b^ ± 7.02	35.80^g^ ± 3.78	20.12 ^b^ ± 2.93
G4: HSO+CNE	114.89 ^c^ ± 10.96	83.09 ^d^ ± 12.68	57.43 ^c^ ± 3.85	40.84 ^f^ ± 3.93	16.75 ^c^ ± 1.63
G5: HSO+VCO+CNE	80.71^h^ ± 9.00	66.97^g^ ± 7.00	22.07 ^f^ ± 5.81	45.25 ^c^ ± 2.65	14.01 ^e^ ± 0.62
G6: HSO+TBHQ	108.79 ^d^ ± 5.86	86.54 ^c^ ± 10.87	50.08 ^d^ ± 5.15	41.40 ^e^ ± 5.56	15.91 ^d^ ± 1.07
G7: VCO Control	84.90 ^f^ ± 9.34	69.25 ^f^ ± 3.84	26.47 ^e^ ± 3.39	44.58 ^d^ ± 3.49	13.12^g^ ± 1.98
G8: CNE Control	86.95 ^e^ ± 4.89	65.01^h^ ± 5.81	28.20 ^e^ ± 3.07	45.75 ^b^ ± 3.57	12.65^h^ ± 1.70

The values are given in terms of mean SD (*n* = 8). Statistically significant differences (*P* < 0.05) based on the Tukey HSD test are denoted by different superscript letters in the same column. TC, total cholesterol; TG, triglycerides; LDL-C, low-density lipoprotein cholesterol; HDL-C, high-density lipoprotein cholesterol; VLDL-C, very low-density lipoprotein cholesterol.

Virgin coconut oil (VCO) (G3) or Curcumin Nano-Emulsion (CNE) (G4) supplementation partially relieved the lipid disorders induced by the HSO, and significant decreases in TC, TG, LDL-C, and VLDL-C, accompanied by significant increases in HDL-C, were observed in comparison to the HSO. Interestingly, the joint VCO and CNE (G5) treatment had the biggest protective effect as it restored the lipid profile to a level comparable to the control groups and was the most improved in all the parameters measured. The TBHQ group (G6) also showed a strong improvement, but not as significant as the effect of the VCO + CNE combination. All in all, the outcomes show that HSO causes serious dyslipidemia, and the provided interventions, especially the VCO+CNE combination, mitigate the changes, which prove the strong synergistic protection effect. Groups that do not have the same letter superscripts vary significantly at *p <* 0.05.

#### 3.4.2 Oxidative stress and antioxidant status

**FIGURE 4:**
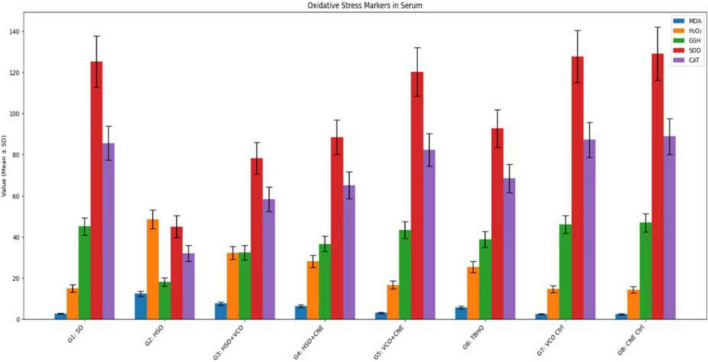
Effect of different treatments on oxidative stress markers in serum of rats.

**FIGURE 5:**
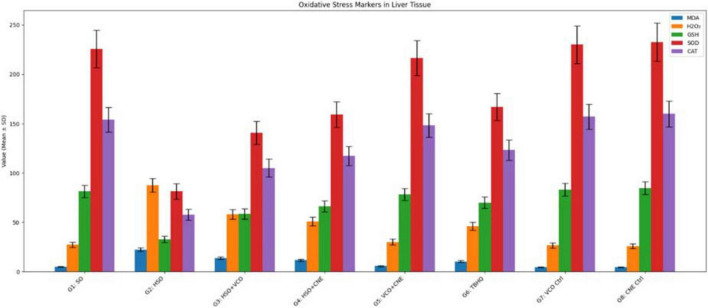
Effect of different treatments on oxidative stress markers in liver tissue of rats.

**FIGURE 6:**
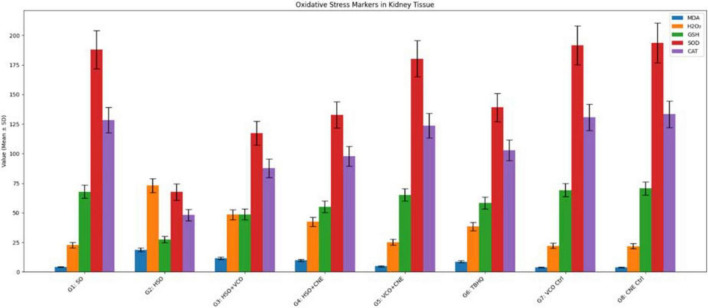
Effect of different treatments on oxidative stress markers in the kidney tissue of rats.

Figures 4–6 show the effect of various treatments on oxidative stress markers on serum, liver, and kidney tissues. The activity of HSO administration (G2) increased considerably the levels of MDA and H_2_O_2_ and reduced those of GSH, SOD and CAT in all the tissues under test in comparison to the control (G1), which showed significant oxidative stress. The antioxidant profile was partially restored with the treatment with VCO (G3) or Curcumin Nano-Emulsion (CNE, G4). The combination treatment of VCO + CNE (G5) was the most effective, where the value of MDA, H_2_O_2_, GSH, SOD, and CAT were successfully normalized back to control. TBHQ (G6) also enhanced antioxidant markers, but not as much as the combination treatment. VCO and CNE control groups (G7 and G8) had a similar antioxidant status as the standard control. These results suggest that HSO causes a great deal of oxidative stress in serum, liver, and kidney, whereas VCO and CNE, especially their combination, produce a great effect of protection due to the increase of enzymatic (SOD, CAT) and non-enzymatic (GSH) antioxidant defenses. All across the experimental groups were found to be statistically significant (*p* < 0.05), as reported in Figures 4–6.

#### 3.4.3 Inflammatory markers

Table 5 shows the relative impact of the various interventions on the inflammatory markers under measure. HSO administration (G2) was associated with a strong inflammatory response, where the highest significant changes (*p* ≤ 0.05) were observed in TNF-α, IL-6, IL-1β, PGE_2_ and CRP as compared to the normal control with cytokine responses changing about 4–5-fold. These increases were significantly reduced by treatment with VCO (G3), CNE (G4) or TBHQ (G6), CNE being stronger than VCO and TBHQ giving moderate effects. The joint action (G5) proved to exhibit the strongest anti-inflammatory activity with all the markers reduced to a level similar to the control and much higher than the effect of individual treatments and TBHQ. These effects were further upheld by grouping in Duncan, G2 was the group with the largest significance letter (a), and the combination group and the control groups were all clustering to the same groups (e–f), making it clear that the inflammatory profile has been nearly normalized. The synergistic VCO+CNE combination was overall better when preventing HSO-induced inflammation as compared to either of the components or the synthetic antioxidant.

**TABLE 5:** Effects of different treatments on pro-inflammatory cytokines and CRP levels in rats exposed to heated sunflower oil (HSO).

Group	TNF-α (pg/mL)	IL-6 (pg/mL)	IL-1β (pg/mL)	PGE2 (pg/mL)	CRP (mg/L)
G1:SO (Control)	45.3^f^ ± 4.5	28.5^f^ ± 3.2	18.7^f^ ± 2.1	125.4^f^ ± 12.5	2.8^f^ ± 0.3
G2: HSO	185.6^a^ ± 18.5	142.8^a^ ± 14.0	95.3^a^ ± 9.5	385.7^a^ ± 38.0	12.5^a^ ± 1.2
G3: HSO+VCO	98.5^b^ ± 9.8	72.3^b^ ± 7.2	52.8^b^ ± 5.3	225.6^b^ ± 22.5	6.8^b^ ± 0.7
G4: HSO+CNE	78.3^c^ ± 7.8	58.5^c^ ± 5.8	42.6^c^ ± 4.3	185.4^c^ ± 18.5	5.2^c^ ± 0.5
G5: HSO+VCO+CNE	48.7^e^ ± 4.9	31.2^e^ ± 3.5	20.5^e^ ± 2.3	132.8^e^ ± 13.0	3.1^e^ ± 0.4
G6: HSO+TBHQ	68.5^d^ ± 6.8	52.3^d^± 5.2	38.4^d^ ± 3.8	175.6^d^ ± 17.5	4.8^d^ ± 0.5
G7: VCO Control	43.8^f^ ± 4.4	27.3^f^ ± 3.0	17.9^f^ ± 2.0	122.5^f^ ± 12.0	2.7^f^ ± 0.3
G8: CNE Control	42.5^f^ ± 4.3	26.8^f^ ± 2.9	17.2^f^ ± 1.9	120.3^f^ ± 12.0	2.6^f^ ± 0.3

Values are mean ± SD (*n* = 8). Different superscript letters within the same column indicates significant differences. At *p* < 0.05 (Duncan’s multiple range test) TNF-α, Tumor Necrosis Factor alpha, IL-6, Interleukin-6, IL-1β, Interleukin-1 beta, PGE2, Prostaglandin E2, CRP, C-reactive protein.

#### 3.4.4 Liver function markers

Table 6 concludes the influence of various interventions on liver functioning scores of rats under the influence of heated sunflower oil (HSO). There was a dramatic increase in the ALT, AST, ALP, and total bilirubin in the HSO group (G2) as compared to the control (G1), which indicated severe damage to hepatocellular and liver dysfunction. Given virgin coconut oil (VCO, G3) or Curcumin Nano-Emulsion (CNE, G4), these increases were significantly decreased, showing partial hepatoprotection. The combination of treatment (HSO + VCO + CNE, G5) had the greatest protective effect, and the enzyme activities and bilirubin levels returned to normal, indicating that VCO and CNE have a strong synergistic effect. The raw material TBHQ (G6) also enhanced liver parameters but not as well as the natural combination. Control groups that were treated with either VCO or CNE only (G7 and G8) were not significantly different than the standard control, which ensured their safety and non-toxicity. These results indicate that the VCO-CNE combination has a better effect in reducing the hepatotoxicity caused by HSO by its antioxidant and anti-inflammatory effects.

**TABLE 6:** Effects of different treatments on liver enzymes and total bilirubin in rats exposed to heated sunflower oil (HSO).

Group	ALT (U/L)	AST (U/L)	ALP (U/L)	Total bilirubin (mg/dL)
G1: SO (Control)	26.23^g^ ± 1.16	86.14^g^ ± 5.87	124.69 ^f^ ± 14.82	0.32 ^f^ ± 0.04
G2: HSO	129.07 ^a^ ± 13.23	291.21 ^a^ ± 18.06	306.01 ^a^ ± 28.59	1.85 ^a^ ± 0.20
G3: HSO+VCO	61.33 ^b^ ± 4.52	152.43 ^b^ ± 17.64	180.59 ^b^ ± 21.49	0.76 ^b^ ± 0.08
G4: HSO+CNE	50.81 ^c^ ± 6.22	119.06 ^c^ ± 7.48	151.51 ^d^ ± 16.69	0.59 ^c^ ± 0.08
G5: HSO+VCO+CNE	31.41 ^e^ ± 2.40	91.97 ^e^ ± 9.44	126.36 ^e^ ± 10.36	0.41 ^e^ ± 0.08
G6: HSO+TBHQ	42.08 ^d^ ± 4.85	117.14 ^d^ ± 11.25	161.89 ^c^ ± 9.49	0.56 ^d^ ± 0.06
G7: VCO Control	29.79 ^f^ ± 3.24	86.81 ^f^ ± 8.67	124.36^g^ ± 11.19	0.31^h^ ± 0.03
G8: CNE Control	26.12^h^ ± 1.26	84.15^h^ ± 7.41	121.86^h^ ± 11.11	0.32^g^ ± 0.05

Values are mean ± SD (n = 8). Different superscript letters within the same column indicate significant differences at *p* < 0.05 (Duncan’s multiple range test) ALT: alanine aminotransferase; AST: aspartate aminotransferase; ALP: alkaline phosphatase.

#### 3.4.5 Kidney function indicators

Table 7 summarizes the effect of different treatments on renal function biomarkers in rats exposed to thermally oxidized sunflower oil (HSO). HSO group (G2) was characterized by a significant increase of urea, creatinine and uric acid as compared to the control group, which indicated the presence of oxidative and inflammatory damage of renal tissue. VCO or CNE (G3 and G4) as an individual supplement did significantly improve these markers, which suggested some nephroprotection. The strongest restoration was achieved with the combined treatment (G5: HSO + VCO + CNE), and the values were close to those of the control group, which confirmed the existence of a strong synergistic effect between VCO and CNE. However, TBHQ (G6) also prevented renal impairment, although it was less effective than the natural combination. G7 and G8 groups (VCO and CNE control groups) showed normal values of renal functioning proving their safety. In general, the findings in Table 7 reveal that VCO-CNE combination provides superior protection against the HSO-induced nephrotoxicity when compared to the individual treatments or synthetic antioxidants.

**TABLE 7:** Effects of different treatments on kidney function in rats exposed to heated sunflower oil (HSO).

Group	Urea (mg/dL)	Creatinine (mg/dL)	Uric Acid (mg/dL)
G1: SO (Control)	41.55^g^ ± 3.40	0.64^g^ ± 0.11	2.73^f^ ± 0.24
G2: HSO	103.61^a^ ± 8.67	1.89^a^ ± 0.18	6.74^a^ ± 0.66
G3: HSO+VCO	63.66^b^ ± 4.96	1.12^b^ ± 0.06	4.13^b^ ± 0.50
G4: HSO+CNE	55.65^c^ ± 4.94	0.96^c^ ± 0.09	3.73^c^ ± 0.38
G5: HSO+VCO+CNE	43.35^e^ ± 3.39	0.72^e^ ± 0.10	2.98^e^ ± 0.14
G6: HSO+TBHQ	49.29^d^ ± 5.45	0.86^d^ ± 0.12	3.26^d^ ± 0.43
G7: VCO Control	41.36^h^ ± 2.71	0.64^f^ ± 0.09	2.48^h^ ± 0.36
G8: CNE Control	42.20^f^ ± 5.43	0.59^h^ ± 0.08	2.60^g^ ± 0.29

Values are mean ± SD (*n* = 8). Different superscript letters within the same column indicate significant differences at *p* < 0.05 (Duncan’s multiple range test).

#### 3.4.6 Histopathology and immunohistochemistry

**FIGURE 7:**
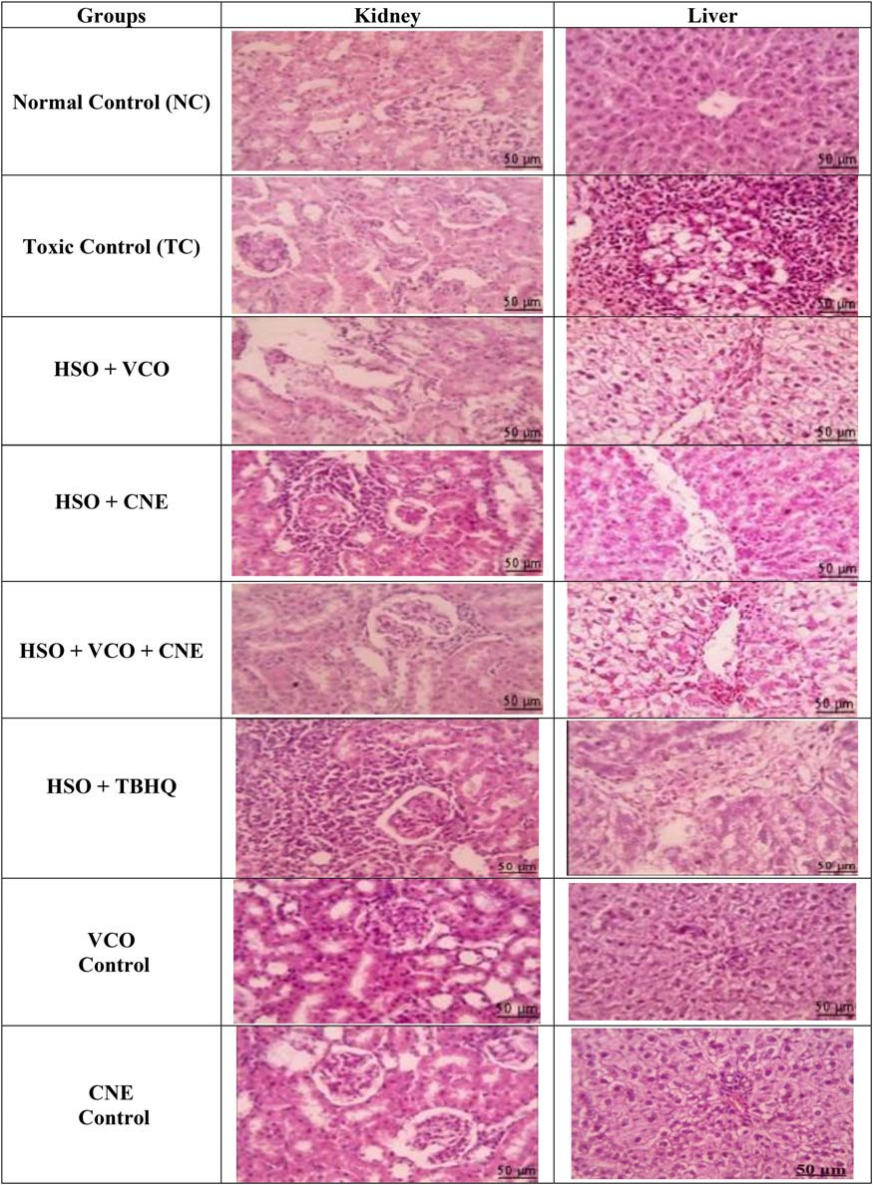
Representative histopathological micrographs of kidney and liver tissues (H&E staining). Representative photomicrographs of kidney (left panel) and liver (right panel) sections from normal control (NC), toxic control (TC), HSO + VCO, HSO + CNE, HSO + VCO + CNE, HSO + TBHQ, VCO control, and CNE control groups. Scale bar = 50 μm.

**FIGURE 8:**
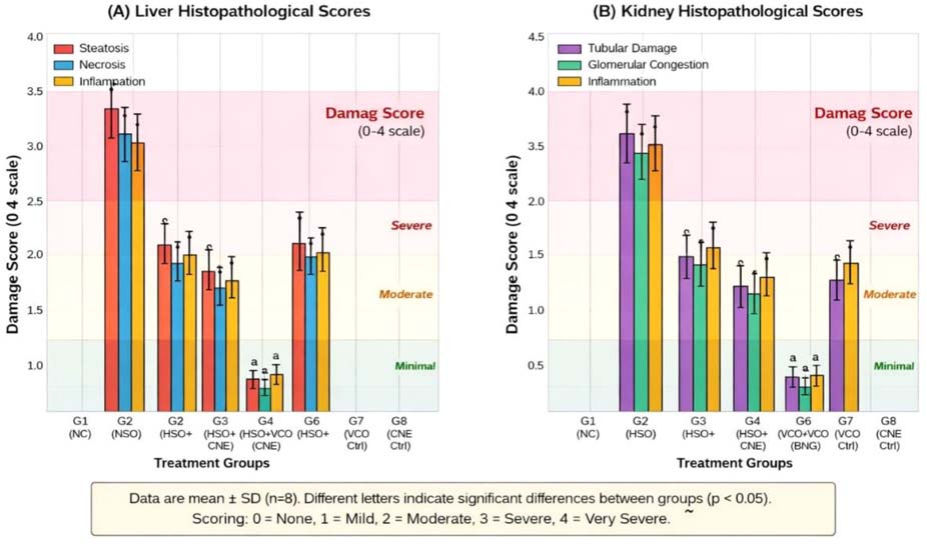
Semi-Quantitative Histopathological Scoring. Bar charts showing average damage scores (0-4 scale) for: **(A)** Liver (steatosis, necrosis, inflammation). **(B)** Kidney (tubular damage, glomerular congestion, inflammation). Data means SD (*n* = 8). Different letters indicate significant differences between groups (*p* < 0.05).

*Histopathology (H&E):* data in Figures 7, 8 showed that the liver sections of the HSO group (G2) showed severe centrilobular necrosis, massive steatosis, and extensive inflammatory cell infiltration, with an average damage score of (3.6 ± 0.3). Kidney sections exhibited acute tubular necrosis and glomerular congestion (damage score: 3.4 ± 0.4). In contrast, the normal control group (G1), as well as the vehicle control groups (VCO control, G7; and CNE control, G8), demonstrated a remarkably well-preserved hepatic and renal architecture with high structural integrity. In these groups, hepatocytes appeared perfectly normal, arranged in regular cords radiating from a clear central vein, with no evidence of congestion, cytoplasmic vacuolation, fatty change, or focal necrosis. Consequently, the damage scores for these groups (G1, G7, and G8) were recorded as 0.00, confirming the safety profile of the individual components of the Nano-emulsion formulation. Furthermore, the synergistic group (G5) showed near-normal hepatic and renal architecture with minimal signs of damage (liver score: 0.4 ± 0.2; kidney score: 0.3 ± 0.1), comparable to the normal control group (G1). The single treatments (G3, G4) and the TBHQ group (G6) showed only partial protection with residual damage (scores ranging from 1.5 to 2.2).

*Immunohistochemistry (IHC):* Quantitative IHC analysis revealed strong positive expression of oxidative damage marker 4-HNE and inflammatory markers NF-κB and TNF-α in the liver and kidney tissues of the HSO group (G2), with area percentages of 45.3 ± 3.2%, 52.1 ± 4.1%, and 48.7 ± 3.8%, respectively. The synergistic group (G5) showed dramatic downregulation of these markers (4-HNE: 8.2 ± 1.5%, NF-κB: 9.1 ± 1.8%, TNF-α: 7.9 ± 1.4%), with expression levels statistically similar to the normal control group (G1) (*p* > 0.05).

Importantly, there was a great increase in the nuclear Nrf2 expression in the synergistic group (G5) (nuclear area percentage: (38.5 ± 3.1%) relative to the HSO group (G2) (12.3 ± 2.1) and even the normal control (G1) (22.1 ± 2.5%), which shows strong activation of the Nrf2 antioxidant pathway. On the other hand, the expression of the nuclear NF-κB in G5 (9.1 + 1.8%) was drastically reduced in comparison to G2 (52.1 + 4.1%) which proves the effective inhibition of the inflammatory cascade.

## 4 Discussion

The current investigation supports the evidence of the positive synergistic effect of antioxidant and anti-inflammatory activity of a Curcumin Nano-Emulsion (CNE) composed of Virgin Coconut Oil (VCO) on the prevention of severe oxidative stress due to the thermal degradation of sunflower oil. We show that not only does this natural mixture better suppress the oxidation of oils compared to the synthetic antioxidant TBHQ but also offers strong protection against the following hepatorenal toxicity by a well-characterized molecular pathway.

Oxidation, phenolics, and color stability in thermally stressed sunflower oil in Figures 4–6. Thermal stress greatly stimulates the oxidative degradation of sunflower oil, as has been previously observed (7) These sustained protection properties are attributed to complementary antioxidant activity of the VCO phenolics and nano-curcumin-based antioxidants, respectively: the radical-scavenging and metal-chelating activity of curcumin-based interventions are enhanced significantly by thermal stress (6). The combination of VCO and CNE has proven to be more effective. The reason is most probably that the systems have complementary antioxidant mechanisms: Phenolics in VCO act as hydrogen donors while nano-curcumin, as a result of enhanced bioavailability, gives strong radical-scavenging and metal-chelating effects (35). The better color stability definitely suggests the restriction of polymerization and pigment degradation as well (36).

### 4.1 Synergistic inhibition of thermal oxidation

Nano-emulsions have been demonstrated to markedly improve curcumin stability, encapsulation efficacy and antioxidant capacity, as demonstrated in quaternized-chitosan coated structures (37) and olive-oil-based nano-emulsions (∼165 nm, > 99% entrapment, 120-day stability) (38) Micro-/nano-carriers have been shown to surmount the inherent limitations of curcumin as a food ingredient Maintaining the stability of the nano-emulsion through this study is vital, as it conserves labile phenolic compounds and volatile bioactive (39) The combination of HSO + VCO + CNE worked together as a team and was more effective than single treatments and TBHQ, resulting in a 62% decrease in total polar compounds by a complex mechanism of physical barrier, complementary radical scavenging, and inhibition of the formation of toxic aldehyde (40, 41) (Figure 2). Reproduced results of nano-based plant extracts to reduce toxic oxidation products through a multifactorial mechanism encompassing physical barriers, complementary radical scavenging, and inhibition of the formation of toxic aldehydes (42).

### 4.2 Molecular mechanisms of *in vivo* protection

The consumption of an oily medium-chain triglyceride (HSO, G2) stimulated moderate dyslipidemia, with increased TC, TG, LDL C, VLDL C and decreased HDL C (Table 4) (43, 44). Virgin Coconut Oil (VCO, G3) and Curcumin Nano-Emulsion (G4) both somewhat corrected these imbalances, possibly through VCO’s medium-chain triglycerides and phenolics, and curcumin’s antioxidant and lipid-modulating effects (45, 46). The combined treatment (VCO + CNE, G5) brought lipid parameters back almost to normal levels, which shows that their joint effect is synergistic (47) It probably demonstrates the co-working of different mechanisms: VCO helping to improve lipid metabolism and reduce oxidation, while curcumin conferring an increased antioxidant and anti-inflammatory effect, thus collectively preventing lipid peroxidation and an increase of lipoproteins (9) The TBHQ group (G6) attained a lesser degree of improvement in the parameters. The results imply that the mix of VCO and nano-curcumin can be an effective natural means of countering dyslipidemia induced by oxidized oils (9, 43).

According to our data (Figures 4–6), the use of heated sunflower oil (HSO, G2) caused the development of intense oxidative stress in serum, liver, and kidney which is evidenced by elevated MDA and H2O2 and lowered SOD, CAT, and GSH. These results are consistent with prior reports suggesting that antioxidant defense is impaired by oxidative stress in a variety of tissues. Interestingly, VCO + CNE treatment (G5) almost completely restored all the oxidative stress markers across tissues, even beyond single treatments and TBHQ (G6), a synthetic antioxidant stabilizer. This type of synergistic activity can probably be described as complementary: VCO as a supplier of membrane-integrated antioxidants and curcumin as a provider of potent radical-scavenging and gene-modulating activity (48).

Combined with the lipid profile results, this combination assists in comprehensive protection on reducing lipid peroxidation, lipoprotein oxidation, and enhancing the lipid metabolism, thereby breaking the vicious loop between oxidative stress and dyslipidemia.

These findings are consistent with previous animal and human studies showing that VCO restores antioxidant enzymes and lowers MDA (49) while curcumin supplementation decreases MDA and increases SOD, CAT, and GSH (9).

Consumption of heated sunflower oil (HSO) triggered a strong inflammatory response, with marked increases in TNF-α, IL-6, IL-1β, PGE2, and CRP compared to control (Table 5).

Supplementation with either Virgin Coconut Oil (VCO) or Curcumin nano-emulsion (CNE) significantly reduced these inflammatory markers, although curcumin showed somewhat stronger effects than VCO alone. The combined VCO + CNE treatment produced the most potent anti-inflammatory effect, normalizing all measured markers—better than each treatment alone or the synthetic antioxidant. These findings are consistent with recent meta-analyses and experimental studies showing that curcumin supplementation lowers CRP, TNF-α, IL-6 levels, and improves antioxidant/anti-inflammatory status, and that VCO exerts anti-inflammatory effects (e.g., reducing cytokines) *in vivo* (46).

Therefore, combining VCO with curcumin (especially in nano-form) appears as a promising natural strategy to counteract inflammation induced by oxidized oils, offering a safer, effective alternative to synthetic stabilizers.

When heated sunflower oil (HSO, G2) was used, a significant hepatocellular dysfunction was observed as evidenced by high levels of ALT, AST, ALP, and total bilirubin (Table 6). Oral administration of either Virgin Coconut Oil (VCO, G3) or nano curcumin extract (CNE, G4) partially returned liver enzyme activities, which proved of moderate hepatoprotection. The combined treatment (VCO + CNE, G5) demonstrated almost the full normalization and was even better than individual treatment and the synthetic antioxidant TBHQ (G6). This implies that the effect is synergistic in hepatoprotection, which is probably through the antioxidant and anti-inflammatory action, including the inhibition of lipid peroxidation, cytokine inhibition and the elevated activity of endogenous detoxifying enzymes (50) Control groups with VCO or CNE alone (G7, G8) had normal enzyme values indicating the safety and non-toxicity of the agents. All of them, in general, support the fact that the VCO, CNE combination is more effective at preventing HSO-induced hepatotoxicity, and its previously noted advantages in lipid profiles, oxidative stress, and inflammation.

Thermal oxidation of sunflower oil (HSO, G2) resulted in a high degree of nephromatotoxicity as evidenced by an increase in the levels of urea, creatinine, and uric acid (Table 7), which is evidence of oxidative and inflammatory harm to the kidney.

The current results indicate that VCO or nano-curcumin alone could only offer partial renal protection against the toxicity induced by HSO (50) However, the combined VCO + CNE treatment induced almost a complete normalization of the renal markers, suggesting an evident synergistic effect, which is better than TBHQ. The complementary mechanisms may be probably behind this synergy: the nano-curcumin stimulates endogenous antioxidant defenses by means of redox-modulating activity (51) and VCO medium-chain fatty acids decrease lipid peroxidation and stabilize cellular membranes.

The conventional renal parameters in the respective control groups ensured the safety of both the agents. This is in line with the recent reports that nano-delivery systems greatly enhance the stability and bioavailability of curcumin and enhance its protective properties (52). Comprehensively, VCO-CNE combination offers a potent and multi-targeted protection against nephrotoxicity caused by HSO.

HSO consumption caused severe systemic toxicity, which is in line with the results of previous research (3, 53). The synergistic treatment (G5) was able to revert these toxic effects to the point of normalizing all the biochemical and biometric parameters. The fact that the synergistic group performed better than TBHQ indicates that their combination delivers in vivo therapeutic effects that are not limited to merely lessening the toxicity of the ingested oil.

The profound protective effect observed in the synergistic group (G5) is mechanistically explained by our quantitative immunohistochemical findings, which provide strong experimental evidence for the proposed dual-pathway modulation. Our results, which show a significant upregulation of nuclear Nrf2 (38.5% area in G5 vs. 12.3% in G2, *p* < 0.001) and dramatic downregulation of nuclear NF-κB (9.1% in G5 vs. 52.1% in G2, *p* < 0.001) (Figure 5), provide compelling evidence that the protective effect is mechanistically driven by a dual modulation of oxidative and inflammatory pathways.

This hypothesis is now strongly supported by our experimental data. The synergistic group was the only treatment that fully restored the depleted endogenous antioxidant enzymes and profoundly suppressed the key pro-inflammatory cytokines TNF-α and IL-6. To ensure a comprehensive evaluation of this approach. While the immunoassay accurately quantified the overall reduction in TNF-α protein levels. Our immunohistochemical (IHC) analysis provided crucial spatial and cellular localization within the tissue architecture. Notably, the quantitative decrease in TNF-α concentration directly mirrored the diminished immunoreactivity and localized expression observed in the tissue sections, particularly within the hepatocytes and renal tubular epithelial cells. This methodology integration confirms that synergistic treatment effectively controls both the absolute production and the localized cellular dissemination of this key cytokine.

**FIGURE 9:**
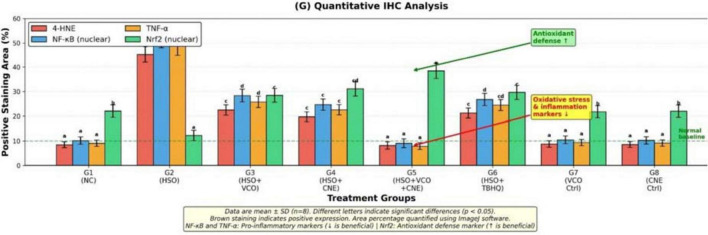
Quantitative IHC analysis. Bar chart represents the percentage of area under the curve of positive staining of 4-HNE, NF-κB, TNF-α, and nuclear Nrf2 in all the treatment groups. Data are mean ± SD (*n* = 8). Different letters indicate significant differences (*p* < 0.05).

The semi-quantitative histopathological scoring (Figures 7, 8) objectively confirms the near-complete tissue protection, in the synergistic G5 with damage scores statistically comparable to the normal control (G1). Importantly, the control groups (G7, G8) exhibited normal architecture (damage score: 0.00), validating the safety of our Nano-emulsion components. Mechanistically, these findings are supported by our quantitative IHC analysis (Figure 9), where the significant reduction in TNF-α and NF-κB and the upregulation of Nrf2 provide clear evidence of dual-pathway modulation, confirming that synergistic treatment effectively controls localized inflammatory dissemination and enhances antioxidant defense (54).

The high nuclear Nrf2 of the synergistic group suggests that the antioxidant response elements (ARE) is strongly transcriptionally activated to induce a strong expression of phase II detoxifying enzymes and antioxidant proteins (55). The high nuclear Nrf2 of the synergistic group suggests that the antioxidant response elements (ARE) is strongly transcriptionally activated to induce a strong expression of phase II detoxifying enzymes and antioxidant proteins (56).

We find excellent agreement with the recent reports that support the role of Nrf2 activation by phytochemicals in suppressing the effects of oxidative stress on various organs (57, 58) and that MCTs can be used to regulate inflammatory signaling and enhance metabolic wellbeing (59) the combination of nano-encapsulated curcumin with VCO is a new and highly efficient approach, which takes advantage of the synergistic action of the two natural compounds.

### 4.3 Study limitations and future directions

Despite the robust experiential design and substantial mechanistic evidence supporting the synergistic protective effects of curcumin nano-emulsion (CNE) and virgin coconut oil (VCO), certain limitations should be acknowledged. The present study was conducted over a relatively short duration; therefore, longer-term investigations are required to assess chronic toxicity, bioaccumulation, and persistence of the observed protective effects.

Additionally, thermal treatment was applied to the oil to allow a precise evaluation of the heat-induced oxidation products. Although this approach offers methodological advantages, it does not fully replicate real-life frying conditions. Accordingly, future studies should focus on oils heated in the presence of food matrices to enhance the relevance of the findings.

Finally, as the results were derived from an experimental animal model, further validation through well-designed clinical trials is required. The integration of metabolomic and proteomic approaches may also provide deeper insights into the molecular mechanisms underlying the observed synergistic effects.

## 5 Recommendations

In the case of the food industry: Consider the use of CNE-VCO blends in commercial frying oils and processed food products to increase oxidative stability, shelf life and safety profile of thermally processed foods. This combination is present in nature and has been proven to be effective, thus making it a viable substitute for artificial antioxidants.

To provide dietary advice: Advocate the use of VCO in domestic food preparation, particularly where it is used with high temperatures, and recommend dietary intake of foods rich in curcumin or well-tested nano-formulations to reduce the health risks of residual toxic compounds in processed and fried foods.

To future research: Carry out well-designed clinical studies to confirm the safety and efficacy of CNE-VCO blend on human population. Explore the chronic disease outcomes of this synergistic mixture (e.g., atherosclerosis, diabetes, non-alcoholic fatty liver disease), and the influence of the combination on the gut-liver axis and systemic inflammation. Further mechanistic studies using advanced omics technologies (metabolomics, proteomics, transcriptomics) will provide deeper insights into the molecular pathways and identify.

## Data Availability

The datasets presented in this study can be found in online repositories. The names of the repository/repositories and accession number(s) can be found in the article/supplementary material.
